# Genetic and Physiological Insights into Salt Resistance in Rice through Analysis of Germination, Seedling Traits, and QTL Identification

**DOI:** 10.3390/life14081030

**Published:** 2024-08-19

**Authors:** Jie Yuan, Qi Wang, Xueying Wang, Bo Yuan, Guojiao Wang, Fengbin Wang, Jiayu Wang

**Affiliations:** 1 Institute of Nuclear and Biological Technologies, Xinjiang Academy of Agricultural Sciences, Urumqi 830091, China; 2 Institute of Agricultural Biotechnology, Jilin Academy of Agricultural Sciences (Northeast Agricultural Research Center of China), Jilin Provincial Key Laboratory of Agricultural Biotechnology, Changchun 130033, China; 3 Rice Research Institute of Shenyang Agricultural University/Key Laboratory of Rice Biology & Genetic Breeding in Northeast China (Ministry of Agriculture and Rural Areas), Shenyang 110866, China

**Keywords:** antioxidant defense, oxidative stress, phenotypic analysis, QTL mapping, salt stress

## Abstract

Understanding the genetic basis of salt resistance in crops is crucial for agricultural productivity. This study investigates the phenotypic and genetic basis of salt stress response in rice (*Oryza sativa* L.), focusing on germination and seedling traits. Under salt stress conditions, significant differences were observed in seed germination and seedling traits between parental LH99 (Indica rice LuHui 99) and SN265 (japonica rice ShenNong 265). Transgressive segregation was evident within the RIL population, indicating complex genetic interactions. Nine QTLs were detected at germination and seedling stages under salt stress, namely *qSGE5* and *qSGE7* for seed germination energy (SGE); *qSGP7* for seed germination percentage (SGP); *qSSH7*, *qSSH9-1*, and *qSSH9-2* for seeding height (SSH); *qSRN6* for root number (SRN); and *qSDW6* and *qSDW9* for dry weight (SDW). Among them, *qSSH9-1* and *qSDW9* were localized in the same interval, derived from the salt-resistant parent SN265. PCA revealed distinct trait patterns under salt stress, captured by six PCs explaining 81.12% of the total variance. PC composite scores were used to localize a QTL associated with early salt resistance in rice *qESC9*, which was located in the same interval as *qSSH9-1* and *qSDW9,* and was subsequently unified under the name *qESC9*, an important QTL for early-growth salt tolerance in rice. Correlation analysis also confirmed a relationship between alleles of *qESC9* and the resistance to salt, underscoring the critical role this locus plays in the determination of overall salt tolerance in rice. Physiological analyses of extreme phenotype lines highlighted the importance of ion exclusion mechanisms in salt-resistant lines, while salt-susceptible lines exhibited elevated oxidative stress and impaired antioxidant defense, contributing to cellular damage. This comprehensive analysis sheds light on the genetic and physiological mechanisms underlying salt stress response in rice, providing valuable insights for breeding programs aimed at enhancing salt resistance in rice.

## 1. Introduction

Salinity stress poses a significant threat to global rice production, limiting yields and threatening food security, particularly in regions where agriculture is dependent on irrigation [1]. Salt stress adversely affects various growth stages of rice, with the seed germination and seedling stages being particularly vulnerable [2]. Understanding the genetic mechanisms underlying salt resistance during these critical phases is imperative for developing resilient rice varieties [3]. 

Salt resistance in rice is a complex trait influenced by the interplay of various genetic and environmental factors [4]. In this context, the identification and characterization of salt resistance loci have garnered significant attention from researchers and agricultural scientists [5]. Lokeshkumar et al. [6] employed 145 rice genotypes, consisting of 100 local varieties and 45 advanced breeding lines gathered from various regions of India. They identified three upland rice varieties in the Saltol region that harbored novel alleles, conferring remarkable salt tolerance to rice. Maniruzzaman et al. [7] identified 12 unique salt tolerance-related QTLs on seven different chromosomes by utilizing a bi-parental mapping population derived from the cross between the salt-sensitive irrigated rice variety BRRI dhan28 and the highly salinity-tolerant Bangladeshi indica rice landrace Akundi. Singh et al. [2] compiled a comprehensive list of 935 reported salt-tolerance QTLs in both rice seedlings and reproductive stages, revealing the chromosomal positions of 63 meta-QTLs that highlight the most crucial genomic regions for salt tolerance in rice. These loci, when understood in depth, have the potential to offer effective strategies for breeding salt-resistant rice varieties and enhancing crop resilience under salinity stress conditions [8].

In recent years, principal component analysis (PCA) has emerged as a widely adopted multivariate data analysis method within the field of biological research [9,10,11]. Its utilization, especially in the context of deciphering the genetic underpinnings of complex traits, has been particularly notable [12]. The integration of PCA with genetic mapping offers distinct advantages by compressing extensive datasets into a few essential dimensions, thereby enhancing our capacity to discern the intricate interplay among various genetic factors. This approach has been successfully demonstrated in prior studies focused on rice, where the simplification of multiple agronomic trait indicators using PCA and the utilization of principal components (PCs) as quantitative metrics have proven to be both practical and insightful [13].

In this study, a Recombinant Inbred Line (RIL) population derived from the cross between the northern super indica rice variety SN265 and the southern japonica rice variety LH99 was utilized as the experimental material. PCA and quantitative trait loci (QTL) analyses were performed for traits related to salt tolerance at the seed germination and seedling stages. Nine QTLs associated with salt tolerance at rice’s germination and seedling stages and one QTL associated with the early salt resistance composite score in rice were localized (see Table 1 for traits). Among them, *qSSH9-1*, *qSDW9*, and, *qESC9* were localized in the same interval, which was identified as an important QTL for salt tolerance in early rice growth, and was uniformly named *qESC9*. Subsequently, we selected two extremely salt-resistant lines carrying the *qESC9* locus from the genetic background of SN265 and two extremely salt-sensitive lines carrying *qESC9* from the genetic background of LH99. Physiological indicators, including reactive oxygen species (ROS) levels and enzyme activities, were measured. The salt resistance traits of the lines carrying *qESC9* from SN265 exhibited significant improvement compared to SN265, LH99, and the salt-sensitive lines. Furthermore, by integrating genetic mapping and QTL localization results, these two superior salt-resistant lines were identified as potential intermediate materials for the genetic enhancement of salt resistance traits in related rice varieties. This research provides valuable insights into the genetic basis of salt resistance in rice, contributing to the development of salt-resistant rice varieties.

## 2. Materials and Methods

### 2.1. Plant Materials

In this study, an RIL population comprising 147 families (F_8_ generation) was developed using the indica rice variety Luhui99 (LH99) as the female parent and the japonica rice variety Shennong265 (SN265) as the male parent.

### 2.2. Salinity Resistance Assessment at the Germination Stage

Following the methodology elucidated by Yang et al. [14], mature seeds from both parents and the RIL population were selected. After breaking dormancy, the seeds were disinfected and rinsed. Subsequently, 50 seeds were placed in 9 cm glass Petri dishes lined with filter paper. Distilled water, 50 mmol L^−1^ NaCl, and 150 mmol L^−1^ NaCl solutions were added separately. The Petri dishes were then incubated in a constant temperature chamber at 30 °C. Germination was defined as the emergence of shoots measuring more than half the length of the seed on the third day. The number of germinated seeds was recorded, and germination energy was calculated using the following formula: Germination Energy (%) = (Number of germinated seeds on day 3/total number of seeds tested) × 100%. On the seventh day, the number of germinated seeds was re-recorded, and the germination rate was calculated using the following formula: Germination rate (%) = (Number of germinated seeds on day 7/Total number of seeds tested) × 100%.

### 2.3. Salinity Resistance Assessment at the Seedling Stage

Seeds from both parents and the RIL population were disinfected and rinsed to break dormancy. The seeds were soaked in darkness at 25 °C for 24 h and then germinated in darkness at 30 °C for 72 h. After germination, 150 seeds with uniform shoot lengths were selected for each line and evenly distributed into three portions. These seeds were sown in a 96-well PCR plate, which was placed inside white plastic trays (35 cm × 27 cm × 10 cm). Distilled water was added to cover the PCR plate. After 2 days of cultivation in a controlled environment chamber, the seedlings were transferred to 1/10 diluted nutrient solution prepared according to the formula provided by the International Rice Research Institute [15]. After 5 days, the seedlings were shifted to a full-strength nutrient solution. At the three-leaf stage, the seedlings were subjected to different concentrations of NaCl (50 mmol L^−1^ and 150 mmol L^−1^) in the nutrient solution. The nutrient solution was replenished twice daily, and every 3 days, the entire solution was replaced. The growth of rice seedlings was observed. On the 14th day, the salt damage levels of the leaves of each line were assessed according to the method of Allen et al. [16]. Five random seedlings from each treatment were selected, and their shoot height, root length, root number, and whole plant dry weight were measured and averaged.

### 2.4. Genetic and QTL Mapping Analysis

We employed 144 polymorphic simple sequence repeat (SSR) and insertion/deletion (Indel) markers, as reported in previous studies [17], which were strategically distributed across the 12 chromosomes to construct a comprehensive genetic map. The QTL analysis was conducted using the inclusive composite interval mapping method, facilitated by the QTL IciMapping 4.0 software [18]. The LOD threshold was set at 2.5, and when the computed LOD value exceeded this threshold, it was deemed that a single QTL exists in that genomic region. Simultaneously, estimates were made for the additive effect values and contribution rates of the identified QTL. The nomenclature of the identified QTL followed the established guidelines outlined by McCouch [19].

### 2.5. Principal Component Analysis

PCA was performed on traits related to salt resistance during germination and seedling stages using GraphPad Prism 9 software, employing a standardized method selection. PCs were chosen based on their eigenvalues. The analysis yielded essential parameters, including eigenvalues (E), the proportion of variance (PV), cumulative proportion of variance (CPV), loadings, and PC scores. Notably, these PC scores were subsequently utilized for QTL localization purposes. To assess the overall performance of each PC, a total score was computed following the formula below:

$$W_{i}=P_{i}/\sum_{i=n}^{n}P_{i}\;(i=1,\;2,\;3,\ldots ,\;n)$$

$$T_{i}=\sum_{i=n}^{n}\left(W_{i}\times S_{i}\right)\;(i=1,\;2,\;,3,\ldots ,\;n)$$

where *W_i_* represents the weight of the *i*th PC. *P_i_* denotes the proportion of variance explained by the *i*th PC. *S_i_* signifies the PC scores of the *i*th lines.

### 2.6. Quantification of Physiological Parameters

The baseline, treatment, and post-boiling conductance values were measured using a DDSJ-308F conductivity meter. Exudation conductivity (%) = (treatment conductivity − baseline conductivity)/(post-boiling conductivity − baseline conductivity) × 100%. The malondialdehyde (MDA) content was determined following the protocol described by Zhang et al. [20]. Optical density values at 532 nm and 600 nm were measured using the thiobarbituric acid method. The determination of hydrogen peroxide content followed the method outlined by Freguson et al. [21]. Hydrogen peroxide was converted into a titanium-peroxide complex using a concentrated HCl solution of T_i_Cl_4_. After multiple washes with acetone and the addition of H_2_SO_4_, the optical density at 410 nm was measured. The measurement of superoxide anion radical content was performed at 530 nm using the hydroxylamine hydrochloride method, following the experimental protocol established by Wang et al. [22]. The sodium ion content in the samples was determined following the method described by Asch et al. [23]. Briefly, the samples were ashed using a mixture of H_2_SO_4_ and H_2_O_2_. The sodium ion concentration was quantified using a flame photometer.

### 2.7. Quantification of ROS Enzyme Activity in Leaves

Leaf samples were homogenized into a uniform paste using ice-cold phosphate buffer, and the supernatant obtained after centrifugation was used as the assay solution. Various physiological enzyme activities were determined: superoxide dismutase (SOD) activity was measured at 560 nm using the nitro tetrazolium blue chloride method described by Giannopolitis and Ries [24]; the peroxidase (POD) activity assay followed the method outlined by Jaffar et al. [25], and changes in absorbance at 470 nm within a continuous 3 min period were determined using the pyrogallol method; the catalase (CAT) activity assay method by Moukette et al. [26] was employed to rapidly measure changes in absorbance at 240 nm within a continuous 3 min period using H_2_O_2_; and the measurement of ascorbate peroxidase (APX) activity followed the protocol established by Ara et al. [27], where the reaction was initiated with ascorbic acid and H_2_O_2_. Rapid changes in absorbance at 290 nm were monitored continuously over 1 min.

### 2.8. Statistical Analysis

All experiments incorporated three biological replicates, and statistical distinctions between samples were assessed using Student’s *t*-test (*p* ≤ 0.05) with GraphPad Prism 9 software.

## 3. Results

### 3.1. Phenotypic Analysis of Rice Germination and Seedling Traits Under Salt Stress 

**Figure 1:**
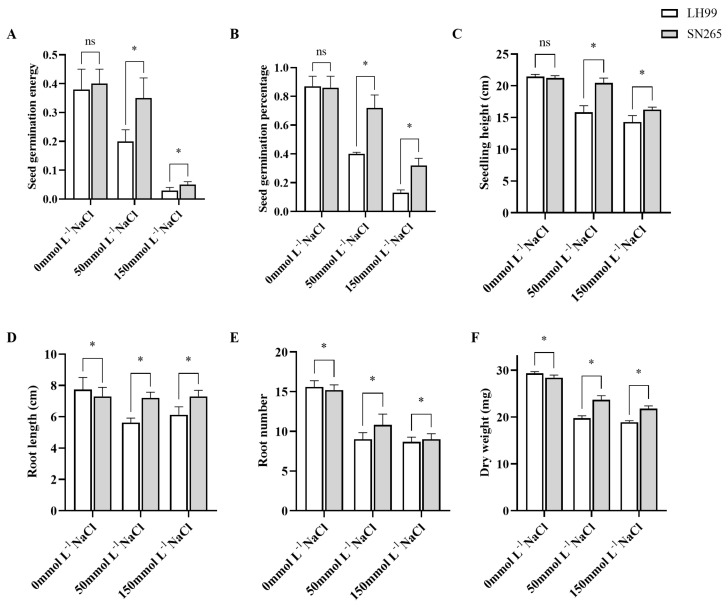
Phenotypic variations in early-stage salt resistance between SN265 and LH99 under varying conditions; (**A**) Seed germination energy; (**B**) Seed germination percentage; (**C**) Seedling height; (**D**) Root length; (**E**) Root number; (**F**) Dry weight; 0 mmol L^−1^ NaCl, 50 mmol L^−1^ NaCl, and 150 mmol L^−1^ NaCl represent control, low salt stress, and high salt stress conditions, respectively. ‘ns’ denotes non-significance, while ‘*’ indicates statistical significance at *p* < 0.05 (Student’s *t*-test).

**Figure 2:**
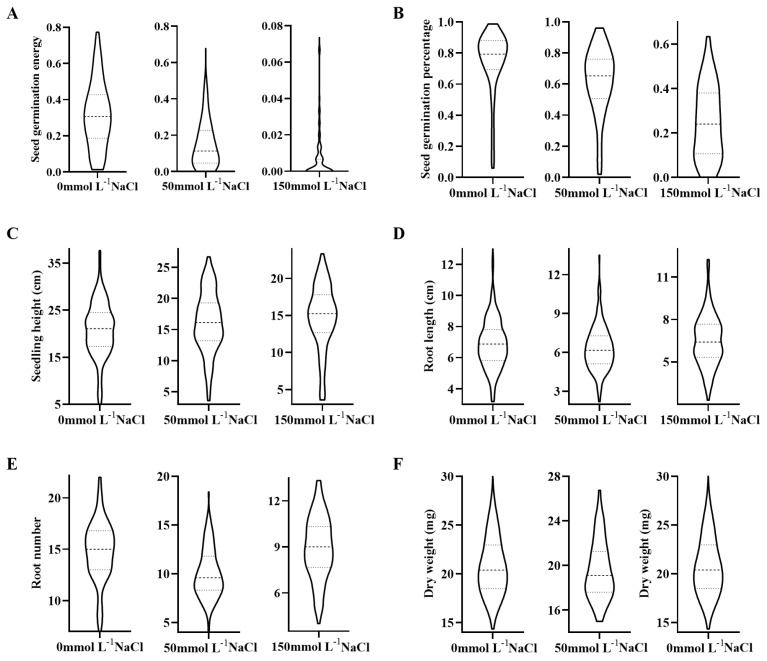
Distribution of phenotypic traits for early-stage salt resistance in a population of 147 RILs derived from hybridization between SN265 and LH99, under various conditions; (**A**) Seed germination energy; (**B**) Seed germination percentage; (**C**) Seedling height; (**D**) Root length; (**E**) Root number; (**F**) Dry weight; 0 mmol L^−1^ NaCl, 50 mmol L^−1^ NaCl, and 150 mmol L^−1^ NaCl represent control, low salt stress, and high salt stress conditions, respectively.

Under non-stress conditions, no significant differences in seed germination energy (C-SGE, which refers to the vigor or strength with which a seed initiates and completes the germination process) and seed germination percentage (C-SGP) were observed between the parents LH99 and SN265. However, under two different concentrations of NaCl salt stress, both parents exhibited significantly reduced germination rates. Particularly noteworthy was SN265, which showed no significant reduction in seed germination energy (L-SGE) and seed germination percentage (L-SGP) under 50 mmol L^−1^ NaCl treatment, while LH99 exhibited a significant decline in germination energy and rate. Under 150 mmol L^−1^ NaCl treatment, both SN265 and LH99 exhibited significantly reduced seed germination energy (H-SGE) and seed germination percentage (H-SGP) (Figure 1A,B). These results indicate substantial differences in germination characteristics, including both genetic variation and salt resistance performance, between SN265 and LH99 under salt stress conditions. Furthermore, within the RIL population, significant variations in germination energy and rate were observed under salt stress, accompanied by apparent transgressive segregation (Figure 2A,B). 

Under non-stress conditions, there were small significant differences in seedling height (C-SSH) between LH99 and SN265 seedling stage; however, significant differences were noted in root number (C-SRN), root length (C-SRL), and dry weight (C-SDW), with LH99 significantly surpassing SN265 (Figure 1C–F). Conversely, under 50 mmol L^−1^ NaCl treatment, seedling height (L-SSH), root length (L-SRL), root number (L-SRN), and dry weight (L-SDW) in LH99 were significantly lower than in the control group. Under 150 mmol L^−1^ NaCl treatment, seedling height (H-SSH), root length (H-SRL), root number (H-SRN), and dry weight (H-SDW) in LH99 were similarly significantly lower than in the control group. In SN265, plant height, root number, and dry weight were significantly lower than in the control, while root length showed no significant difference from that in the control group. Under salt stress, significant differences in seedling traits were observed between SN265 and LH99, particularly in salt resistance. Similarly, within the RIL population under salt stress, all four traits exhibited significant transgressive segregation, with the population’s average values higher than those of the parent LH99, but lower than those of the paternal parent SN265 (Figure 2C–F).

### 3.2. PCA of Rice Germination and Seedling Traits under Salt Stress

In PCA, the information is considered representative when the cumulative proportion of variance of the PCs exceeds 80%. In this study, the eigenvalues corresponding to the top six PCs were all greater than 1.0, explaining 31.68%, 13.55%, 12.05%, 10.62%, 7.27%, and 5.95% of the total variance, respectively. The cumulative proportion of variance for these top six PCs was 81.12%, indicating that these mutually independent components encapsulated 81.12% of the information pertaining to the salt stress traits studied.

**Figure 3:**
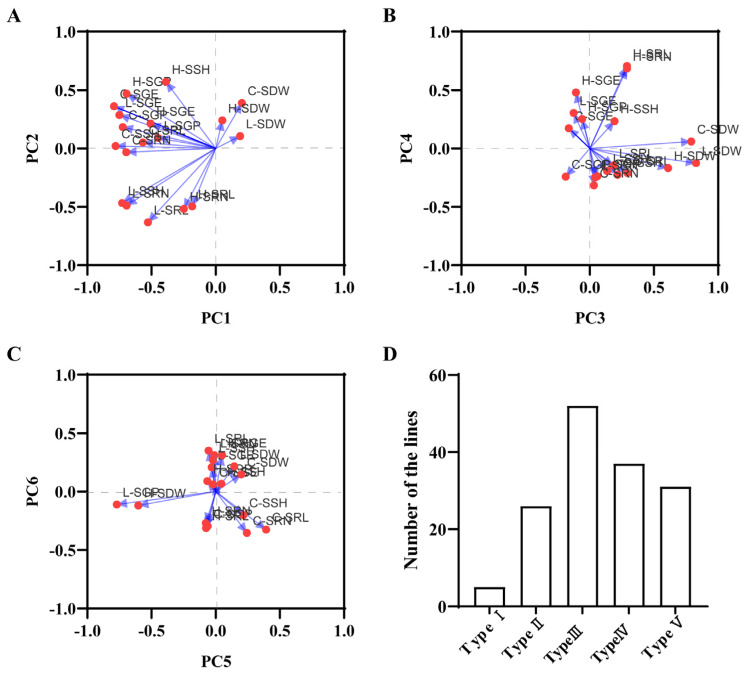
Distribution of loadings for various traits within the top six principal components (PCs); The distribution of five typological strains based on aggregate PC scores in the RIL population under diverse conditions. (**A**) Distribution of loadings for various traits in PC1 and PC2; (**B**) Distribution of loadings for various traits in PC3 and PC4; (**C**) Distribution of loadings for various traits in PC5 and PC6; (**D**) Distribution of the five types in the RIL population.

The PC analysis revealed distinct patterns among the salt resistance traits. The first PC was characterized by a relatively strong positive loading for C-SDW (0.203) and substantial negative loadings for C-SGE (−0.793), C-SSH (−0.781), L-SGE (−0.751), L-SSH (−0.729), and C-SGP (−0.725), collectively defining the ‘seed germination energy and seedling height’ factor. The second PC exhibited notable positive loading for H-SSH (0.570) and substantial negative loadings for L-SRL (−0.633), H-SRN (−0.518), and H-SRL (−0.497), collectively characterizing the ‘seedling height under high salt stress and seedling root length under low salt stress’ factor (Figure 3A). Moving on, the third PC was marked by high positive loadings for L-SDW (0.827), C-SDW (0.790), and H-SDW (0.61), with C-SGP (−0.188) displaying a notable negative loading, encapsulating the ‘seedling dry weight under salt stress’ factor. In the fourth PC, significant positive loadings were observed for H-SRL (0.705), H-SRN (0.683), H-SGE (0.478), and SBV (0.451), while C-SRN (−0.317) had substantial negative loadings, defining the ‘root length and root number of seedlings under high salt stress’ factor (Figure 3B). The fifth PC exhibited marked positive loading for C-SRL (0.392) and negative loadings for L-SGP (−0.771) and H-SDW (−0.603), characterizing the ‘seed germination rate under low salt stress’ factor. The sixth PC was characterized by a strong positive loading for L-SRL (0.350) and a notable negative loading for C-SRL (−0.326), referred to as the ‘root length of seedlings under low salt stress’ factor (Figure 3C).

### 3.3. QTL Analysis for Salt Resistance-Related Traits during Seed Germination and Seedling Stages

Nine QTLs associated with rice seedling and germination stages were detected on chromosomes 5, 6, 7, and 9 under salt stress. The phenotypic contribution of these QTLs ranged from 7.10% to 10.16% (Table 2). Among them, *qSGP7*, with the highest phenotypic contribution, was located between RI05304-RM11 on chromosome 9, with a LOD value of 3.77, and an allele from SN265. *qSSH9-1* and *qSDW9* were co-localized on the same interval RM3700-RM7424 on chromosome 9, with LOD values of 2.52 and 2.92, and phenotypic contributions of 8.75% and 8.59%, respectively, with alleles from SN265. 

### 3.4. QTL Analysis of PC Composite Scores for Salt Resistance-Related Traits during Seed Germination and Seedling Stages

**Figure 4:**
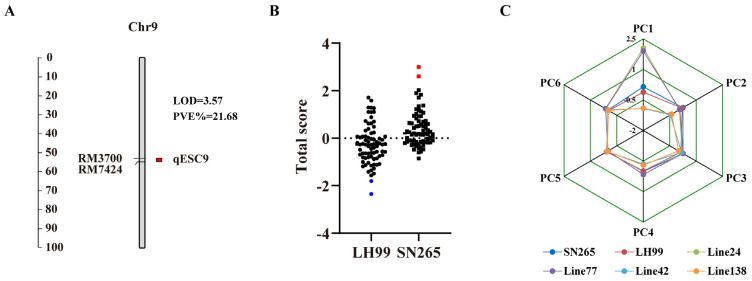
Correlation analysis of qESC9 localization with integrated salt tolerance score and identification of extreme phenotypic lines. (**A**) Location on the genetic map, LOD scores, and contribution rates of the detected qESC9 associated with composite scores for early-stage salinity tolerance in rice; (**B**) distribution of salinity tolerance composite scores in the RIL population harboring *qESC9* alleles from different parental sources; (**C**) comparison of scores across six principal components for parents SN265 and LH99 and four extreme lines. Of these, line 24 and line 77 were extremely salt-resistant lines, and line 42 and line 138 were extremely salt-susceptible lines.

Using the composite scores derived from the six PCs, the RILs were collectively assessed based on their respective weights, resulting in a score distribution spanning from −2.34 to 2.60. These lines were subsequently categorized into five distinct groups according to their total scores, which were organized in a hierarchical manner, reflecting the salt resistance traits from the most superior to the least favorable (Figure 3D). Through QTL mapping, we identified a QTL locus, designated as *qESC9*, associated with the early salt resistance composite score in rice. This locus was situated on chromosome 9. The position is the same as *qSSH9-1* and *qSDW9*, with a LOD score of 3.57 and a contribution of 21.68% to the phenotypic variance. The enhancing allelic variant of *qESC9* was derived from SN265 (Figure 4A). 

### 3.5. Correlation Analysis of qESC9 with Salt Resistance Composite Score and Identification of Extreme Phenotype Lines

Our genetic mapping efforts confirmed the presence of the *qESC9* locus on chromosome 9. To validate the correlation between *qESC9* alleles and the salt resistance composite score, independent phenotypic analysis was performed on the RIL population. The results demonstrated a consistent pattern, indicating that the SN265 allelic variant at the *qESC9* locus exhibited a significant correlation with elevated salt resistance composite scores (Figure 4B). This discovery underscores the direct impact of specific genetic variations on the overall salt resistance phenotype in rice. Notably, extreme phenotypes exhibited conspicuous allelic variations at this locus, highlighting its pivotal role in determining salt resistance levels (Figure 4C).

### 3.6. Analysis of Salt Resistance Physiological Indices in Extreme Phenotype Lines

**Figure 5:**
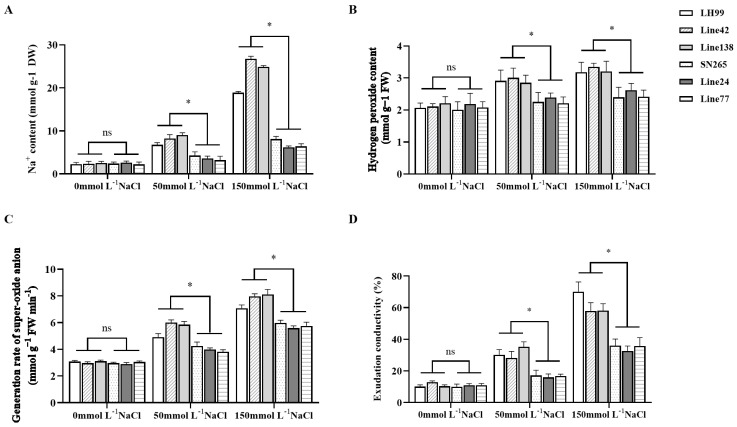
Comparison of ion content, hydrogen peroxide levels, superoxide anion generation rate, and extracellular conductivity among parental lines, highly salt-tolerant genotypes, and extremely salt-sensitive genotypes during rice seed germination and seedling stages under salinity stress conditions at the major salt resistance locus *qESC9*. (**A**) ion content; (**B**) hydrogen peroxide levels; (**C**) superoxide anion generation rate; (**D**) extracellular conductivity. Of these, line 24 and line 77 were extremely salt-resistant lines, and line 42 and line 138 were extremely salt-susceptible lines. respectively. ‘ns’ denotes non-significance, while ‘*’ indicates statistical significance at *p* < 0.05 (Duncan’s Multiple Range Test).

**Figure 6:**
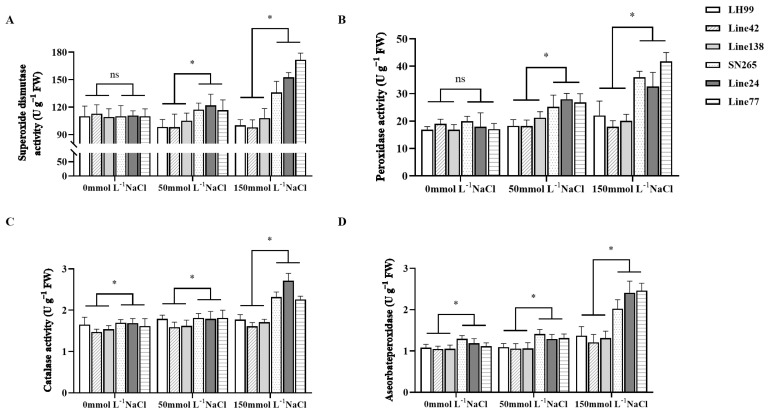
Comparison of SOD, POD, CAT, and APX among parental lines, highly salt-tolerant genotypes, and extremely salt-sensitive genotypes during rice seed germination and seedling stages under salinity stress conditions at the major salt resistance locus *qESC9*. (**A**) Superoxide dismutase activity; (**B**) Peroxidase activity; (**C**) Catalase activity; (**D**) Ascorbate peroxidase activity. Of these, line 24 and line 77 were extremely salt-resistant lines, and line 42 and line 138 were extremely salt-susceptible lines. ‘ns’ denotes non-significance, while ‘*’ indicates statistical significance at *p* < 0.05 (Duncan’s Multiple Range Test).

We selected the parental SN265 and LH99, as well as the extremely salt-resistant lines 24 and 77, and the extremely salt-susceptible lines 42 and 138, for physiological parameter analysis to elucidate distinct physiological response patterns in extreme phenotype lines. In comparison to the salt-susceptible lines, the highly salt-resistant lines exhibited a significant capacity to maintain lower sodium ion accumulation in their tissues (Figure 5A). This phenomenon suggests the existence of an efficient ion exclusion mechanism in these resistant lines, preventing the toxic buildup of sodium ions in plant cells. Furthermore, the salt-susceptible lines displayed elevated levels of cell membrane permeability and oxidative stress markers, including increased hydrogen peroxide, superoxide anion content, and exudation conductivity (Figure 5B). These results indicate that these lines are susceptible to salt-induced oxidative stress, resulting in cellular oxidative damage (Figure 5C,D). The increased activity of antioxidant enzymes, including SOD, POD, CAT, and APX, in these lines signifies an attempt to counteract oxidative damage. However, the capacity of these susceptible lines to scavenge reactive oxygen species appears to be insufficient, leading to cellular damage and impairing growth and development (Figure 6A–D).

## 4. Discussion

The increasing threat of salinity stress on global rice production necessitates a deep understanding of the genetic and physiological mechanisms underlying salt resistance. This study delves into the intricate interplay of genetic factors influencing salt resistance during the crucial germination and seedling stages of rice.

### 4.1. qESC9 Plays a Crucial Role in Salinity Resistance in Rice

Salt tolerance in rice is a crucial trait for ensuring stable production in saline environments [4]. Developing salt-tolerant rice varieties is essential in addressing the challenges posed by soil salinity. Recent advancements in scientific research have enabled the identification of key QTLs associated with salt tolerance in rice [28]. For instance, Nayyeripasand et al. [29] employed genome-wide association studies (GWASs) and identified novel QTLs on chromosome 1 that significantly enhance rice’s tolerance to salt stress. Additionally, Theerawitaya et al. [30] conducted a study focusing on ion homeostasis and identified multiple QTLs associated with sodium and potassium uptake, providing valuable insights into the physiological mechanisms underlying salt tolerance in rice. The complexity of the genetic network involved in salt tolerance is well documented [28]. QTLs governing multiple traits related to salt tolerance often cluster in specific chromosomal regions [5]. The identification of these genetic loci provides a promising avenue to enhance rice’s adaptability to saline conditions, demonstrating the potential for improving rice salt tolerance through genetic means [31].

**Figure 7:**
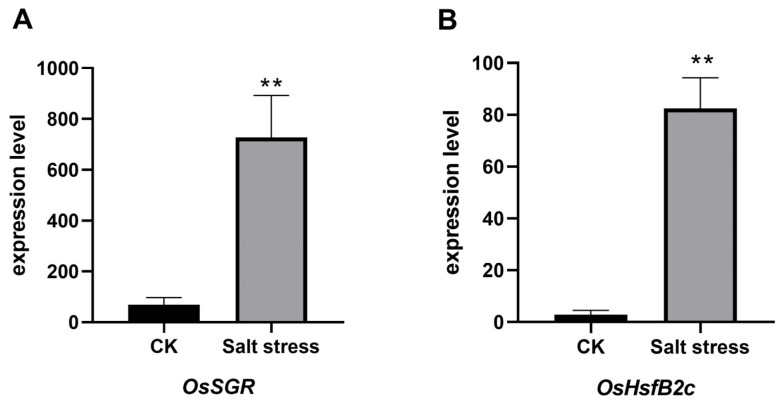
*OsSGR* and *OsHsfB2c* in the GSE6901 gene data microarray expression level under salt stress. (**A**) *OsSGR* expression level; (**B**) *OsHsfB2c* expression level. ‘**’ indicates a significant difference, *p* < 0.01 (Student’s *t*-test).

In this study, the transgressive segregation observed within the RIL population highlights the complex genetic interactions underlying salt resistance traits, indicating the involvement of multiple loci in the salt stress response. Notably, the identification of the *qESC9* locus on chromosome 9 as a significant determinant of early salt resistance composite scores in rice underscores the pivotal role of specific genetic loci in conferring salt resistance. The presence of this locus in the salt-resistant parent SN265 and its positive correlation with elevated salt resistance composite scores reaffirm the importance of *qESC9* in determining overall salt resistance. The identification of extreme phenotype lines further emphasizes the impact of allelic variations at this locus on salt resistance levels. Compared to previous investigations, we identified seven genes associated with salt tolerance in the *qESC9* locus. *Os9BGlu33* stands out as a key regulator of rice seed germination, root elongation, and drought tolerance, simultaneously participating in the signal transduction of auxin and abscisic acid [32]. Mutants of *OsITPK6* exhibit lower tolerance to osmotic stress, providing evidence for the role of *OsITPK6* in regulating rice osmotic stress tolerance [33]. *OsFes1C*, as the nucleotide exchange factor for *OsBiP1*, is involved in endoplasmic reticulum and salt stress responses, and overexpression of *OsFes1C* enhances salt tolerance in plants [34]. *OsDREB1A* and *OsDREB1B*, two genes among the identified candidates, have demonstrated stress-responsive characteristics, with overexpression conferring drought, high-salt, and cold tolerance in Arabidopsis and tobacco, respectively [35,36]. *OsUGE3*, a positive regulator of cellulose and hemicellulose biosynthesis, enhances biomass, mechanical strength, and salt tolerance by increasing polysaccharide deposition in the cell wall [37]. *OsPRR95*, acting as a transcriptional regulator, promotes seed germination and seedling growth by inhibiting abscisic acid signaling, induced by ABA, salt, and mannitol treatments [38]. Then, we screened two candidate genes, *OsSGR* and *OsHsfB2c*, whose expression was significantly up-regulated in *qESC9* using the rice seedling salt tolerance gene data chip GSE6901 in the gene expression database (GEO) (Figure 7A,B). *OsSGR* may be involved in regulating or directly mediating PaO activity and modulating reactive oxygen species metabolism [39]. *OsHsfB2c* is a heat shock transcription factor that is a central regulator in heat stress defense response [40]. Both *OsSGR* and *OsHsfB* affect rice resistance to abiotic stresses to some extent. Collectively, our study shows that *qESC9* plays a key role in salinity tolerance in rice. 

### 4.2. Implications for Breeding Programs

Understanding the intricate genetic basis and physiological mechanisms of salt tolerance in rice is essential for targeted breeding programs [41]. The identification and characterization of specific QTLs provide promising avenues for developing rice varieties that can thrive in salt environments [42]. In this study, the integration of PCA with QTL mapping has proven instrumental in deciphering the complex nature of salt resistance traits. PCA allowed for the categorization of traits into distinct components, enhancing our understanding of the relationships among different salt resistance indicators. The subsequent QTL analysis based on PCA-derived composite scores led to the identification of the *qESC9* locus, providing a holistic view of the genetic factors influencing salt resistance. Additionally, understanding the physiological responses of extreme phenotype lines provides valuable clues for *qESC9* improving antioxidant defense mechanisms in susceptible varieties. By leveraging these findings, breeding programs can expedite the development of salt-resistant rice cultivars, thereby ensuring food security in regions vulnerable to salinity stress.

## 5. Conclusions

In this study, nine QTLs associated with salt tolerance at the germination and seedling stages of rice and one QTL associated with the early integrated score in rice were localized, and the *qESC9* locus was identified as a key genetic factor for salt tolerance at the germination and seedling stages of rice. This highlights the complex genetic architecture of salt tolerance and the differing physiological responses under stress. Salt-resistant lines demonstrate effective ion management, contrasting with the oxidative stress in susceptible lines. These insights are crucial for breeding salt-tolerant rice varieties, and addressing global food security challenges in saline environments. In future research, by combining these QTL mapping results, we will use backcrossing to aggregate excellent traits and achieve resistance improvement in rice.

## Figures and Tables

**Table 1:** Measurement of correlated traits and trait abbreviations.

Trait	Abridge	Conditions (mmol L^−1^ NaCl)	Abridge
Seed germination energy	SGE	0	C-SGE
		50	L-SGE
		150	H-SGE
Seed germination percentage	SGP	0	C-SGP
		50	L-SGP
		150	H-SGP
Seedling height	SSH	0	C-SSH
		50	L-SSH
		150	H-SSH
Root length	SRL	0	C-SRL
		50	L-SRL
		150	H-SRL
Root number	SRN	0	C-SRN
		50	L-SRN
		150	H-SRN
Dry weight	SDW	0	C-SDW
		50	L-SDW
		150	H-SDW

**Table 2:** QTL localization for salt resistance-related traits during seed germination and seedling stages.

Traits	Salt Stress Intensity	Chr.	QTL	Location Interval	LOD Value	Add.	Var. (%)	Source of Potentiating Allele
Seed germination energy	1	5	*qSGE5*	RM519-RM413	2.78	0.04	8.08	SN265
	1	7	*qSGE7*	PSM142-RI05304	3.31	0.05	8.54	SN265
Seed germination percentage	2	7	*qSGP7*	RI05304-RM11	3.77	0.07	10.16	SN265
Seedling height	3	7	*qSSH7*	RM1186-RM6835	3.30	−1.54	8.60	LH99
	3	9	*qSSH9-1*	RM3700-RM7424	2.52	1.56	8.75	SN265
	4	9	*qSSH9-2*	RM7424B-STS18	3.16	1.72	9.45	SN265
Root number	4	6	*qSRN6*	RI02763- RI04969	2.64	0.65	8.02	SN265
Dry weight	4	6	*qSDW6*	RI02729-RM454	2.57	−1.86	7.10	LH99
	4	9	*qSDW9*	RM3700-RM7424	2.92	2.01	8.59	SN265

Note: 1 is rice treated with 50 mmol L^−1^ NaCl at seed germination, 2 is rice treated with 150 mmol L^−1^ NaCl at seed germination, 3 is rice treated with 50 mmol L^−1^ NaCl at seedling stage, and 4 is rice treated with 150 mmol L^−1^ NaCl at seedling stage.

## Data Availability

All relevant data are provided as figures or tables within the paper.
